# A case of small cell neuroendocrine carcinoma of the ampulla of Vater

**DOI:** 10.1186/s40792-020-00915-9

**Published:** 2020-06-26

**Authors:** Hiroharu Ito, Yoshiyuki Wada, Yuko Takami, Tomoki Ryu, Hiroki Ureshino, Hajime Imamura, Shin Sasaki, Akihisa Ohno, Masayuki Hijioka, Toyoma Kaku, Ken Kawabe, Shigeto Kawauchi, Hideki Saitsu

**Affiliations:** 1 Department of Hepato-Biliary-Pancreatic Surgery, Clinical Research Institute, National Hospital Organization Kyushu Medical Center, 1-8-1 Jigyohama, Chuo-ku, Fukuoka, 810-8563 Japan; 2 Department of Gastroenterology, National Hospital Organization Kyushu Medical Center, Fukuoka, Japan; 3 Department of Pathology, National Hospital Organization Kyushu Medical Center, Fukuoka, Japan

**Keywords:** Neuroendocrine carcinoma, Neuroendocrine neoplasm, Neuroendocrine tumor, Ampulla of Vater, Bile duct

## Abstract

**Background:**

Gastroenteric neuroendocrine carcinomas (NECs) account for 6.2% of gastroenteric neuroendocrine tumors (NETs), and only 1% or less of gastroenteric NETs occur in the ampulla of Vater (AoV). Clinical features of NEC of the AoV remain obscure.

**Case presentation:**

A 65-year-old man visited a general practitioner because of jaundice, and an abdominal contrast-enhanced computed tomography scan revealed a tumor of 11 mm in diameter, which was enhanced in the arterial phase at the duodenal papilla, with dilation of the upstream bile duct. Gastrointestinal scope revealed an unexposed tumor of the AoV. Based on a biopsy of the site, a moderately differentiated tubular adenocarcinoma was suspected, and pancreatoduodenectomy was performed. Histopathological examination revealed dysplasia and highly proliferative small tumor cells, with solid and nodular formation at the AoV. Histological analysis showed a high mitotic count, and immunohistochemical staining revealed a Ki-67 index of 40–50% and cells positive for synaptophysin, chromogranin A, and p53. Small cell-type NEC was finally diagnosed. Four months post pancreatoduodenectomy, multiple liver metastases developed, and systemic chemotherapy was administered. Salvage liver resection for liver metastases was performed 14 months after the pancreatoduodenectomy. Unfortunately, multiple liver metastases developed 2 months after liver resection, and the patient died 18 months after the pancreatoduodenectomy.

**Conclusions:**

Neuroendocrine carcinoma originating from the bile duct is very rare; therefore, in this article, we provide a review of the literature and a case report.

## Background

According to the Clinical Practice Guidelines for Gastroenteropancreatic Neuroendocrine Neoplasms (GEP-NEN) 2019, pancreatic and gastrointestinal neuroendocrine tumors are relatively rare, with three to five new patients per 100,000 population per year [1]. In the World Health Organization (WHO) 2010 classification, tumors derived from neuroendocrine cells were collectively referred to as neuroendocrine tumors (NETs) and were classified into NET grade (G)1, NET G2, and neuroendocrine carcinoma (NEC) according to their reproductive activity (i.e., their mitotic count and Ki-67 index), not according to their histology.

In the past, NECs were defined by a high mitotic count (over 21/10 in a high-power field (HPF)) or with a Ki-67 index exceeding 20%. However, the Ki-67 index was found to be high even in well-differentiated NETs. Therefore, these cases came to be referred to as NET G3 and are distinct from NECs, which are poorly differentiated and have a Ki-67 index of more than 20%.

Subsequently, criteria for NET G3 and NECs were defined for pancreatic NEN in the WHO 2017 classification. The classification was changed by the clinical findings that pancreatic NET G3 showed a low response to combination chemotherapy containing platinum-based regimen but had a relatively good prognosis. On the other hand, pancreatic NEC had successfully responded to combination chemotherapy containing platinum-based regimen, but its prognosis was very poor [2, 3].

Furthermore, this new classification was adopted to the gastroenteric NEN in the WHO 2019 classification. Gastroenteric NECs account for 6.2% of gastroenteric NETs in Japan, and only 1% or less of gastroenteric NETs occur in the ampulla of Vater (AoV). Here, we report a very rare case of neuroendocrine carcinoma of the AoV.

## Case presentation

**Fig. 1:**
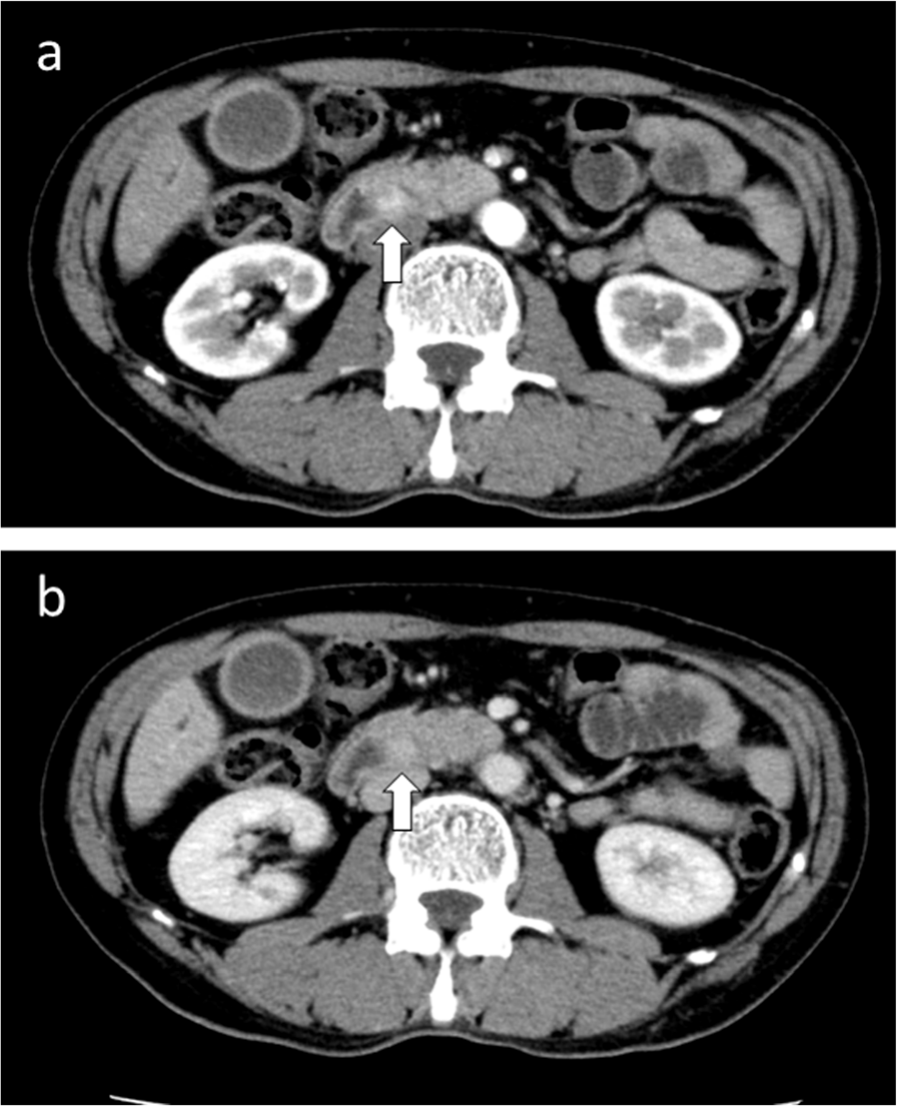
Abdominal contrast-enhanced CT scan image. **a** The tumor of 11 mm in diameter at the duodenal papilla showed enhancement in the arterial phase (white arrow). **b** The enhancement of the tumor lasted until the portal phase

**Fig. 2:**
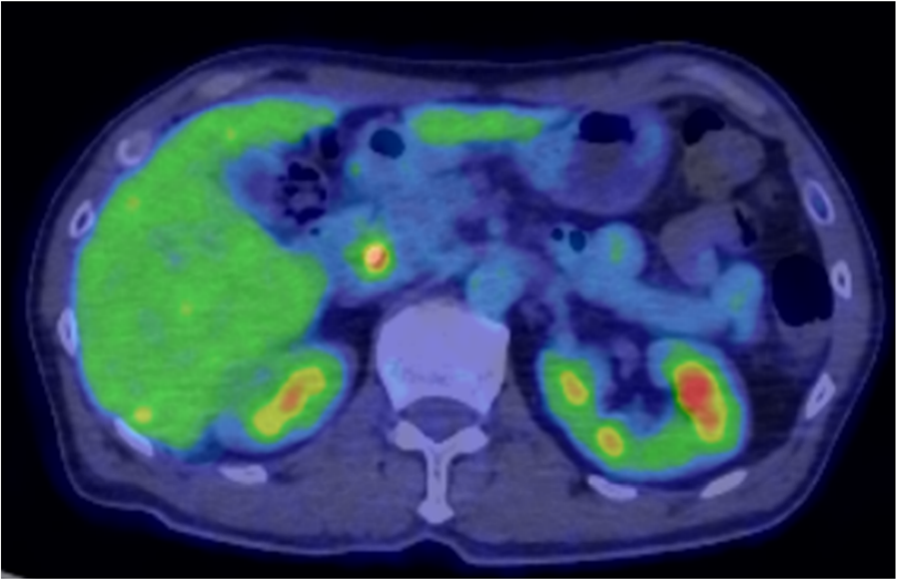
Positron emission tomography-computed tomography scan image. The tumor exhibited uptake of fluorodeoxyglucose

**Fig. 3:**
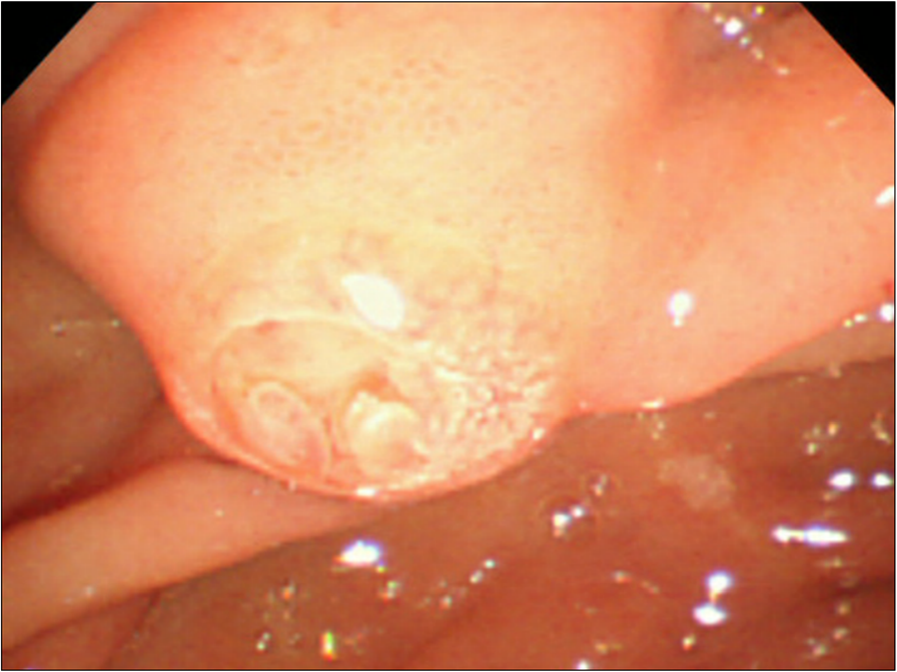
Endoscopic retrograde cholangiopancreatography showed an unexposed tumor at the papilla of Vater

**Fig. 4:**
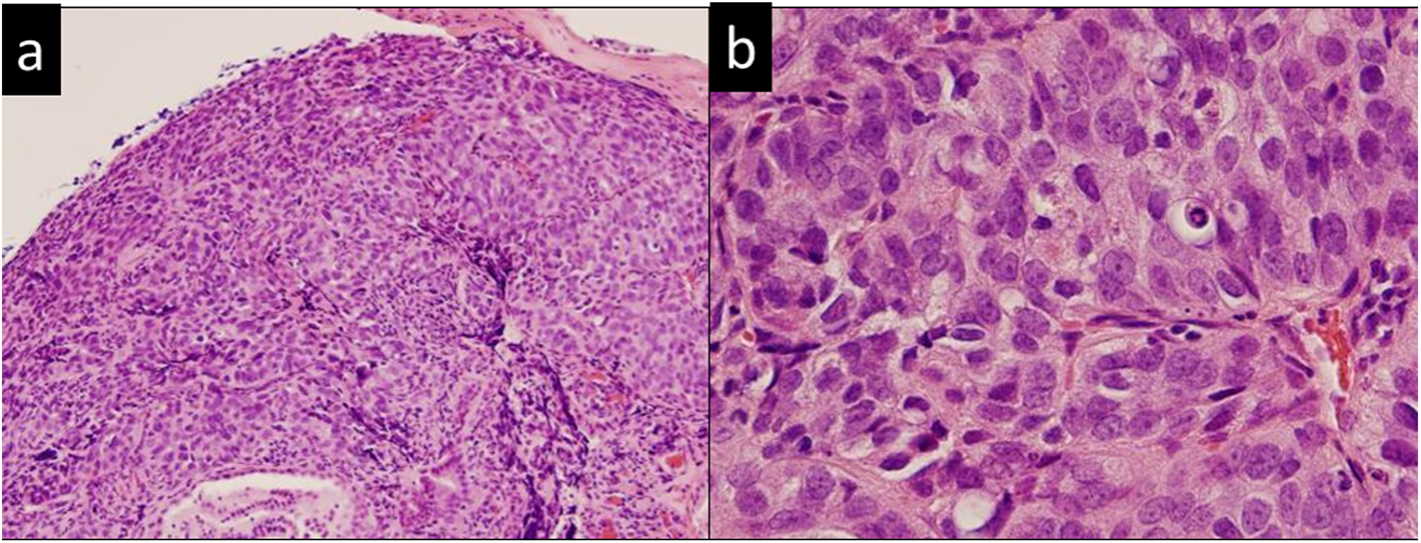
Histopathological findings of the biopsy specimen of the tumor. **a** Low-power field. **b** High-power field

A 65-year-old male patient visited a general practitioner because of jaundice and was referred to our hospital. Laboratory results showed elevated levels of total bilirubin, transaminases, and biliary enzymes (total bilirubin 12.6 mg/dL, direct bilirubin 8.6 mg/dL, aspartate aminotransferase 48 IU/L, alginate aminotransferase 44 IU/L, gamma-glutamyl transpeptidase 84 IU/L, alkaline phosphatase 999 IU/L). Tumor markers were all negative (carcinoembryonic antigen 4.0 ng/mL, carbohydrate antigen 19-9 2.0 U/mL, DUPAN-2 < 25 U/mL, Span-1 15 mg/dL). An abdominal contrast-enhanced computed tomography (CT) scan showed that a tumor of 11 mm in diameter was enhanced in the arterial phase and in the delayed phase at the AoV (Fig. 1a, b). The upstream common bile duct and intrahepatic bile duct were dilated. No distant metastasis or lymph node metastasis was found. Magnetic resonance imaging showed that the tumor of the AoV exhibited high intensity on diffusion-weighted images, low intensity on T1-weighted images, and high intensity on T2-weighted images. Positron emission tomography revealed that fluorodeoxyglucose was accumulated with a maximum standardized uptake value of 4.5 at the tumor of the AoV (Fig. 2). Endoscopic retrograde cholangiopancreatography showed an unexposed tumor of the AoV (Fig. 3) and stenosis of the lower bile duct with dilatation of the upstream bile duct. A biopsy specimen of the site showed proliferating tumor cells in the lamina propria mucosae, partially with a glandular structure (Fig. 4a, b). A moderately differentiated tubular adenocarcinoma was suspected.

**Fig. 5:**
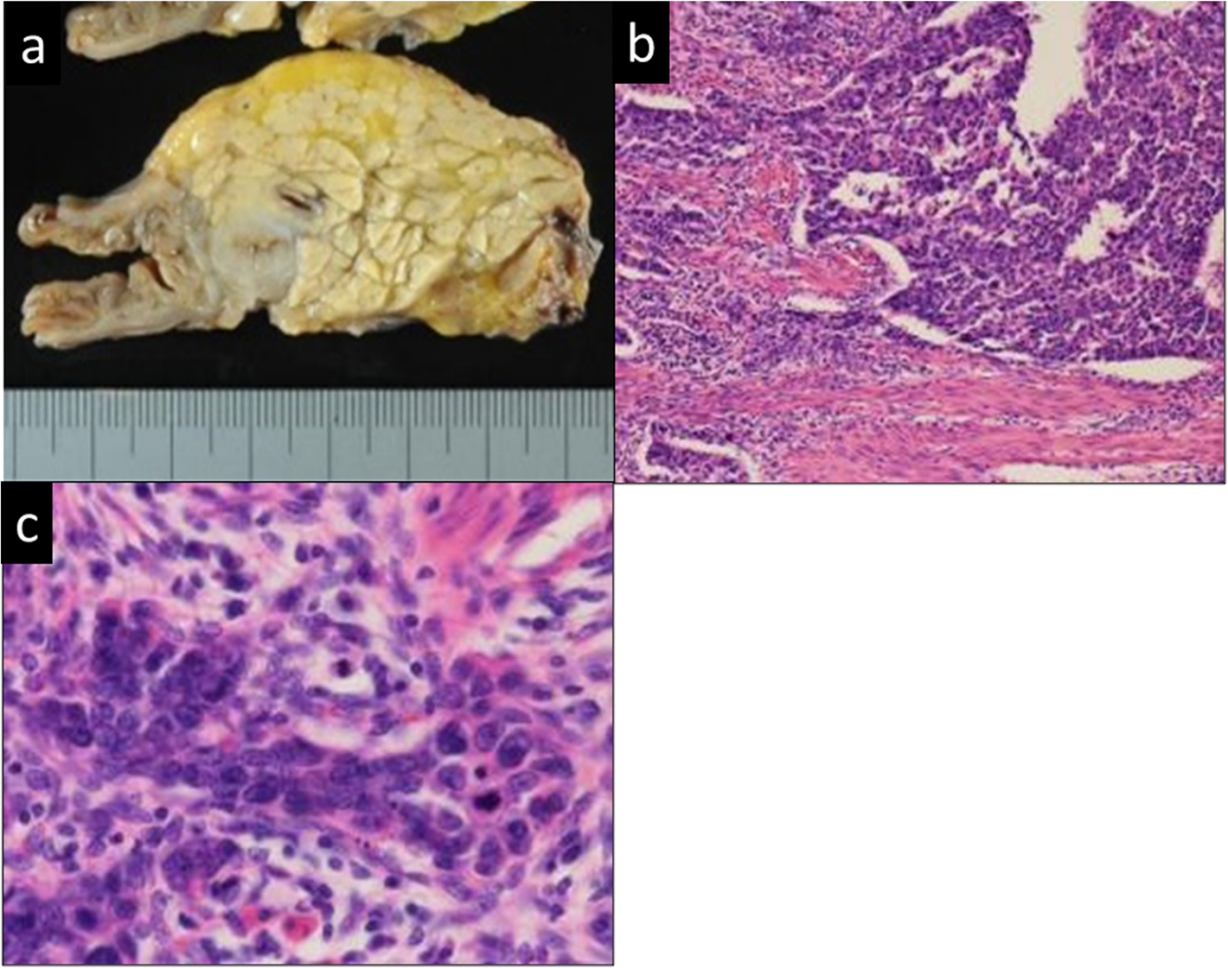
Postoperative histopathological examinations. **a** Macroscopic findings. **b** Low-power field. **c** High-power field

**Fig. 6:**
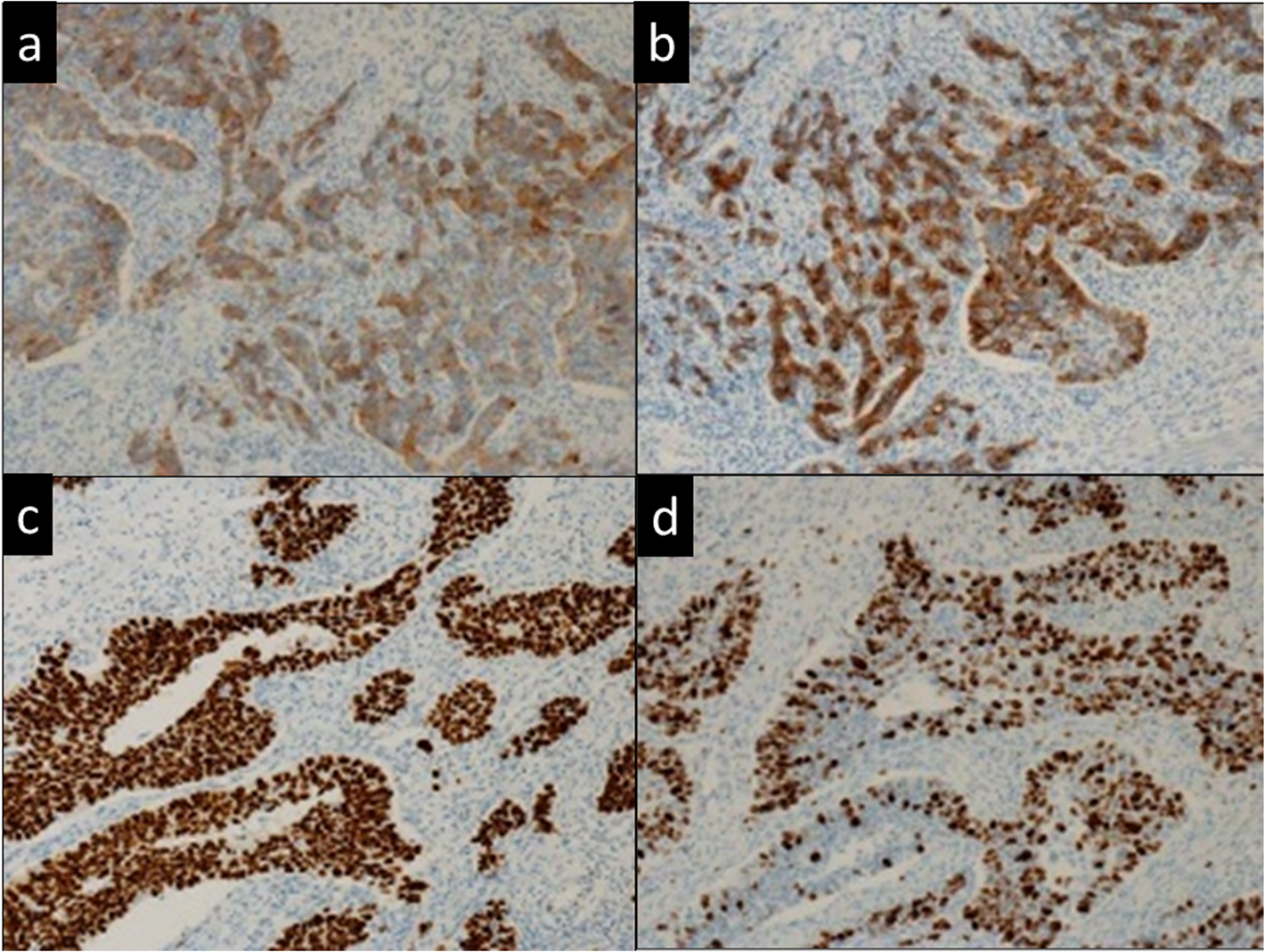
Immunohistochemical findings. **a** Synaptophysin stain. **b** Chromogranin stain. **c** Ki-67 stain. **d** p53 stain

Subtotal stomach-preserving pancreatoduodenectomy and D2 lymph node dissection were performed with a diagnosis of duodenal papilla carcinoma. Macroscopic findings showed a grayish-white solid tumor at the AoV (Fig. 5a). Histopathological examination showed that small monotonous tumor cells, with high-grade dysplasia, proliferated arranged in cords, nest, or tubular structures along with fibrous stroma (Fig. 5b, c). A glandular structure such as adenocarcinoma was not evident. The tumor cells were found to have invaded the muscle propria and the mucosal layer of the duodenum. The mitotic count was 30–40/10 in HPF. Immunohistochemical staining showed cells positive for neuroendocrine cell markers, synaptophysin, and chromogranin A. The Ki-67 index was found to be 40–50% (Fig. 6a, b). Moreover, p53 staining was positive for more than 95% of cancer cells (Fig. 6c), indicative of p53 overexpression. Rb1 staining was positive for about 70% of cancer cells (Fig. 6d). Lymph node metastasis was not evident in the resected lymph nodes. Based on the above findings, a small cell-type NEC was finally diagnosed.

**Fig. 7:**
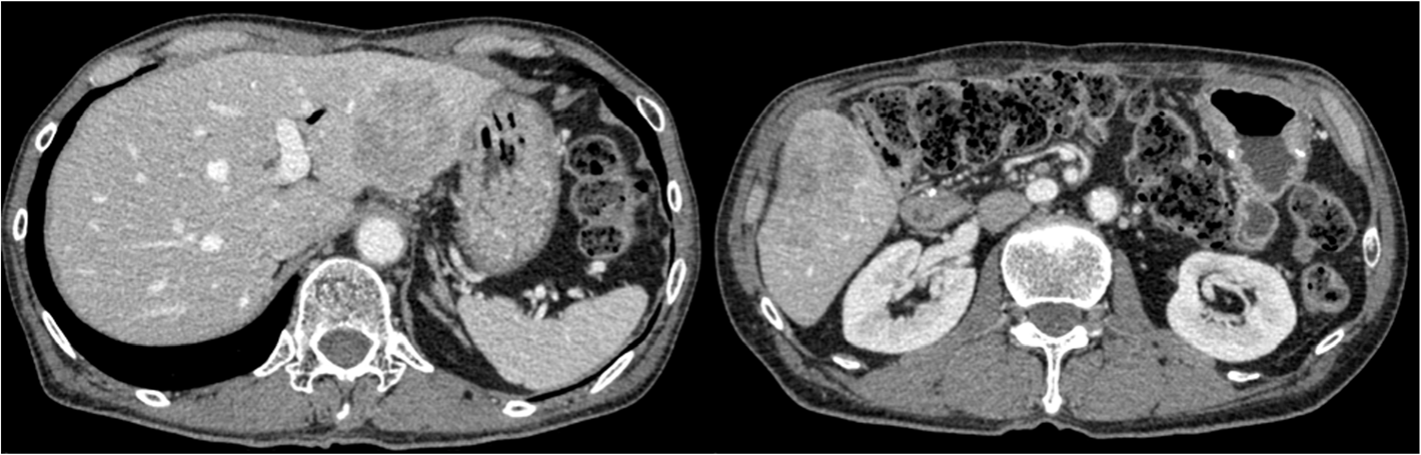
Abdominal contrast-enhanced CT scan image at the hepatic resection after chemotherapy showing growth of colorectal liver metastases (**a**, **b**)

Four months after pancreatoduodenectomy, an abdominal CT scan showed multiple liver metastases, and a percutaneous liver biopsy was performed, which confirmed metastasis of the NEC. Five months after the operation, chemotherapy consisting of a combination of irinotecan (CPT-11) and cisplatin (CDDP) was administered. The best response to the chemotherapy was stable disease. However, multiple liver metastases progressed after 8 cycles of chemotherapy (Fig. 7a, b), and no new liver metastasis developed. Salvage liver resection for liver metastases was performed 14 months after the pancreatoduodenectomy. Unfortunately, multiple liver metastases developed 2 months after liver resection. Combination chemotherapy of etoposide and CDDP was administered, but it was ineffective. The patient died 18 months after the pancreatoduodenectomy.

## Discussion

This is a case report of very rare small cell NEC of the AoV. Although NENs or NECs of the pancreas have been frequently reported, these are rarely found in the biliary tract. Of the NENs found in the biliary tract, the gall bladder and the ampulla of Vater are common locations [4, 5]. In a Chinese case study [5], NECs were found to account for 85.8% of all biliary tract NEN cases. This is much higher than that reported in a Surveillance, Epidemiology, and End Results (SEER) study (47.7%) [4]. Moreover, the SEER study reported that NECs of the AoV were found in 47 (2.3%) out of 2024 gastrointestinal NEC patients [4]. Furthermore, small cell NECs of the AoV were found in only 7 patients (1.6%) out of 443 gastrointestinal small cell NEC patients.

Patients with biliary tract NEC were reported to have a median age of 50–60 years and more likely be male (82.4%) [5–7]. Ten case reports of small cell NEC of the AoV have been reported, and 8 of these 10 patients were male [6, 7]. In contrast, the SEER study [4] reported that small cell NECs of the gall bladder and extrahepatic bile duct were more common in females, indicating a possible ethnic difference between Asian and Western populations.

The differentiation between NENs and NECs is important because they require different treatments. However, their differentiation using radiological examination is ultimately difficult. Moreover, radiological examinations generally fail to distinguish NENs or NECs from other bile duct cancers because of their overlapped imaging appearance, which contributes to the high rate of misdiagnosis [8]. In fact, this case, which was misdiagnosed as an adenocarcinoma of the biliary tract preoperatively, was postoperatively diagnosed as NEC of the AoV by histopathological and immunohistochemical findings. Postoperative histopathological findings of the resected tumor showed a nested solid and nodular tumor, which constituted of small tumor cells. Furthermore, immunohistochemical findings showed an overexpression of synaptophysin and chromogranin A, indicative of a neuroendocrine tumor such as NEN or NEC, and not an adenocarcinoma. Moreover, a high mitotic count (30–40/10 HPF) and a high Ki-67 index (40–50%) were suggestive of NET G3 or NEC. In addition, the presence of small, poorly differentiated tumor cells with a high rate of morphological atypia implied NEC to be more likely. Immunohistochemical staining of p53 and Rb1 is also considered useful for definitive diagnosis of NEC [3, 9]. In this case, overexpression of p53 supported a definitive diagnosis of NEC. On the other hand, over half of NECs display an absence of Rb1-positive cells [3, 9]; however, this case showed an overexpression of Rb1.

The characteristics of NEC include diffuse overexpression of p53 protein and diffuse loss of Rb1 protein, and these immunostainings are extremely useful for distinguishing NEC from NET G3. Konukiewitz et al. [9] reported that abnormal nuclear p53 and Rb1 staining was found in 29/37 and 22/37 poorly differentiated neuroendocrine neoplasms, respectively, whereas all well-differentiated neuroendocrine neoplasms showed normal p53 and Rb1 expression. Hijioka et al. [3] reported that NET G3 showed no abnormal Rb1 expression (0%), whereas NEC G3 showed Rb1 loss (54.5%). Yachida et al. [10] reported that abnormal immunolabeling patterns of p53 and Rb1were frequent (p53, 18 of 19, 95%; Rb, 14 of 19, 74%) in NECs of the pancreas, whereas p53 and Rb1 immunolabeling was intact in PanNETs. As described in these reports [3, 9, 10], abnormal expression of p53 or loss of Rb1 is certainly useful for diagnosis of NEC; however, this pattern of expression is not necessarily found in all NECs. When comparing p53 and Rb1, p53 is considered more useful for dedifferentiation between NET G3 and NEC.

In this case, a diagnosis of papilla carcinoma was suggested preoperatively based on the biopsy specimen of moderately differentiated adenocarcinoma. Retrospective analysis of the preoperative biopsy specimen after the surgery revealed that the tumor was composed of small cell carcinoma cells, without an adenocarcinoma component. NEN that occurs in the biliary tract is often associated with an adenocarcinoma, and it is thought that neuroendocrine tumors may appear prior to an adenocarcinoma developing [8, 11]. A biliary tract NEN is difficult to distinguish from biliary tract cancer using diagnostic imaging, because of early deep staining on contrast-enhanced CT [11]. A biopsy is often a false negative because the tumor takes on the form of a submucosal tumor, so a definitive diagnosis before surgery is not easy [8]. There was difficulty in providing a preoperative diagnosis in this case as contrast-enhanced CT scan showed early deep staining and an adenocarcinoma was suspected based on a biopsy specimen. The patient was finally diagnosed with NEC after surgery. If NEC was diagnosed preoperatively, chemotherapy, and not surgery, would have been the considered treatment, as prognosis of NEC, even after complete resection, is very poor [6]. The Japanese guidelines for the management of pancreatic and gastrointestinal tract NECs recommend chemotherapy instead of surgery [1]; however, at the time of diagnosis of this case, these guidelines were not yet published.

Surgical indications and chemotherapy for NET G3, a well-differentiated tumor, and NEC, a poorly-differentiated tumor, differ in the treatment strategy for pancreatic and gastrointestinal neuroendocrine tumors, so the distinction between NET G3 and NEC is important. Pancreatic and gastrointestinal tract NEC is high grade of malignancy and has an extremely poor prognosis, especially with distant metastases [12]. In the future, it is hoped that optimal chemotherapy will be established in conjunction with the accumulation of cases.

Salvage resection for metastatic NEC is controversial; therefore, chemotherapy should be considered first. In this case, the size of multiple liver metastases marginally increased following chemotherapy, but their number did not increase. Moreover, the tolerability of chemotherapy decreased owing to bone marrow suppression. Therefore, the patient and the attending physician were anxious for resection. Following the discussion of the institutional hepato-biliary-pancreatic cancer board with the multidisciplinary team at our institution, hepatic resection for multiple liver metastases was permitted as a challenging treatment for this case. During decision making, it was taken into consideration that the number of liver metastases did not increase after chemotherapy and that the multiple liver metastases could be completely resected.

Generally, the prognosis of small NEC is very poor because early metastasis to the liver and regional lymph nodes is common [6]. The SEER study [4] reported that the 5-year survival rate was 8% for small cell NEC of the gall bladder and 0% for small cell NEC of the extrahepatic bile duct. The Chinese case study [5] reported that the 2-year survival rate for G1 and G2 NENs was 100%, whereas it was 55.6% and 66.7%, respectively, for NECs and mixed adenoneuroendocrine carcinomas. In the 10 case reports published [6, 7], rapid recurrence was often reported, and most patients died within 2 years, except for one patient who presented with long-term disease-free survival.

A limitation of this study was that we were unable to analyze the expression of somatostatin receptor (especially SSTR2), ATRX, DAXX, and KRAS, as well as other markers, which are also considered useful for discriminating immunohistochemical and genetic findings between NET G3 and NEC. However, the facilities that can perform these analyses are limited; therefore, we were unable to perform these in this case.

## Conclusion

NEC originating from the bile duct is very rare; therefore, in this article, we report a case of small cell NEC of the ampulla of Vater. The prognosis of this cancer is very poor, even if complete resection is performed. Surgical indication for NEC may be extremely limited, if NEC can be preoperatively diagnosed.

## Data Availability

Data will be made available from the corresponding author upon request.

## Abbreviations

NEC: Neuroendocrine carcinoma
AoV: Ampulla of Vater
GEP-NEN: Gastroenteropancreatic neuroendocrine neoplasms
WHO: World Health Organization
NET: Neuroendocrine tumor
G: Grade
CT: Computed tomography
